# Association of maternal ethnicity and urbanicity on severe pediatric disease: a nationwide cohort study

**DOI:** 10.1186/s12887-019-1885-9

**Published:** 2019-12-23

**Authors:** Ya-Ting Chang, Huei-Shyong Wang, Jia-Rou Liu, Chi-Nan Tseng, I-Jun Chou, Shue-Fen Luo, Chang-Fu Kuo, Lai-Chu See

**Affiliations:** 1 Department of Pediatrics, Chang Gung Memorial Hospital at Linkou, Taoyuan, Taiwan; 2 Department of Molecular Medicine and Surgery, Karolinska University Hospital, Karolinska Institutet, Stockholm, Sweden; 3 Department of Public Health, College of Medicine, Chang Gung University, Taoyuan, Taiwan; 4 Department of Cardiac Surgery, Chang Gung Memorial Hospital at Linkou, Taoyuan, Taiwan; 5 Division of Rheumatology, Allergy and Immunology, Chang Gung Memorial Hospital at Linkou, Taoyuan, Taiwan; 6 Biostatistics Core Laboratory, Molecular Medicine Research Center, Chang Gung University, Taoyuan, Taiwan; 7 Center for Big Data Analytics and Statistics, Chang Gung Memorial Hospital at Linkou, Taoyuan, Taiwan

**Keywords:** Severe diseases, Children, Transnational marriage, Urban–rural

## Abstract

**Background:**

A healthy migrant effect on birth outcomes has been reported, however, whether this protective effect persists throughout childhood is unknown. The effect of urbanicity on child health among an immigrant population is unclear. The objective of this study was to compare the incidence rate and cumulative incidence of severe diseases among urban children of Taiwan-born mothers, rural children of Taiwan-born mothers, urban children of foreign-born mothers, and rural children of foreign-born mothers.

**Methods:**

A nationwide cohort study was conducted for children born in Taiwan during 2004–2011 and follow-up till age 4 to 11 years old by linkage the Taiwan Birth Registry 2004–2011, Taiwan Death Registry 2004–2015, and National Health Insurance Research Database 2004–2015. Cox proportional hazards model (multivariable) was used to examine differences among the four study groups.

**Results:**

There were 682,982 urban children of Taiwan-born mothers, 662,818 rural children of Taiwan-born mothers, 61,570 urban children of foreign-born mothers, 87,473 rural children of foreign-born mothers. Children of foreign-born mothers had a lower incidence of vasculitis, mainly Kawasaki disease. The incidences of congenital disorders did not differ between children of foreign-born mothers and children of Taiwan-born mothers. The incidence of psychotic disorders was higher in urban children. However, children in rural areas had a higher incidence of major trauma/burn and a higher mortality rate.

**Conclusions:**

A healthy migrant effect was only seen for Kawasaki disease. The mental health of urban children born to immigrant mothers warrants concern.

## Background

The increasing frequency of transnational marriage and migration has made the health of children of foreign-born mothers (FBMs) an important public health concern. Children born to FBMs appear to be healthier than those born to native-born mothers [1], and the “healthy migrant theory” attempts to explain this epidemiologic paradox [2]. A systematic review and meta-regression analyses found that black migrant women had a lower risk of delivering low-birth-weight (LBW) and preterm birth babies than did US-born black women. Hispanic migrant women also had better birth outcomes, but Asian and white migrants did not. As compared with the respective native-born populations, Sub-Saharan African and Latin-American/Caribbean women had a higher risk of delivering LBW babies in Europe but not in the US, and South-central Asians had worse birth outcomes in both continents. These findings indicate that the association between migrant status and adverse birth outcomes varies by migrant suburban status and is sensitive to the definitions used for the migrant and reference groups [3].

In Taiwan, the number of transnational marriages progressively increased from 1998, peaked in 2003 (28.0%), and gradually decreased thereafter (11.1% in 2012) [4], because of the complex dynamics nature of mobility factors (such as economic, political, racial) in Taiwan and nearby Southeast Asia and mainland China [5]. The proportion of babies born to FBMs to all babies born was 11.5 to 13% in 2004–2006 and slowly decreased to 7.5% in 2012 [4]. Transnational marriages primarily involved Taiwanese men married to women from Southeast Asia or mainland China and were arranged by marriage brokers [6]. The Taiwanese men in transnational marriages are predominantly of undesirable partners (elderly veterans, divorcees, the disabled, farmers, or working-class Men) [5]. Previous studies found that the incidences of preterm birth (< 37 weeks of gestational age) [7–10], LBW (< 2500 g) [8–11], and neonatal mortality (death during the first 10 days of life) [9, 12] were significantly lower for the newborns of FBMs than for those of Taiwan-born mothers (TBMs). However, the rate of orofacial cleft among newborns did not significantly differ between these groups [13].

Few studies have investigated differences in outcomes among children of FBMs and TBMs. Pediatric health may be affected by urbanization level, implying varied accessibility of medical resources and social support. We examined whether the healthy immigrant effect persists throughout childhood and how urbanization level affects the health of children born to immigrant and native-born women. Incidences of severe pediatric diseases until age 11 years were compared among the children of urban TBMs, rural TBMs, urban FBMs, and rural FBMs.

## Methods

### Source of data

We analyzed data from the Taiwan Birth Registry (TBR) 2004–2011, Taiwan Death Registry (TDR) 2004–2015, and National Health Insurance Research Database (NHIRD) 2004–2015. The nationality of the mother has been recorded in TBR since 2004. The NHIRD is the health insurance claims database of the Taiwan National Health Insurance (NHI) system. In 1995, Taiwan launched a single-payer NHI system, which has covered 99% of the population of the country since 2014 [14]. The databases contain registration and original claims data for reimbursement, including demographics characteristics, dates of clinical visits, medical diagnoses, medical expenditure, and details of prescriptions, examinations, and procedures. The TBR, TDR, and NHIRD are linked by a unique personal ID number, which is de-identified for research purposes.

### Study design

**Fig. 1:**
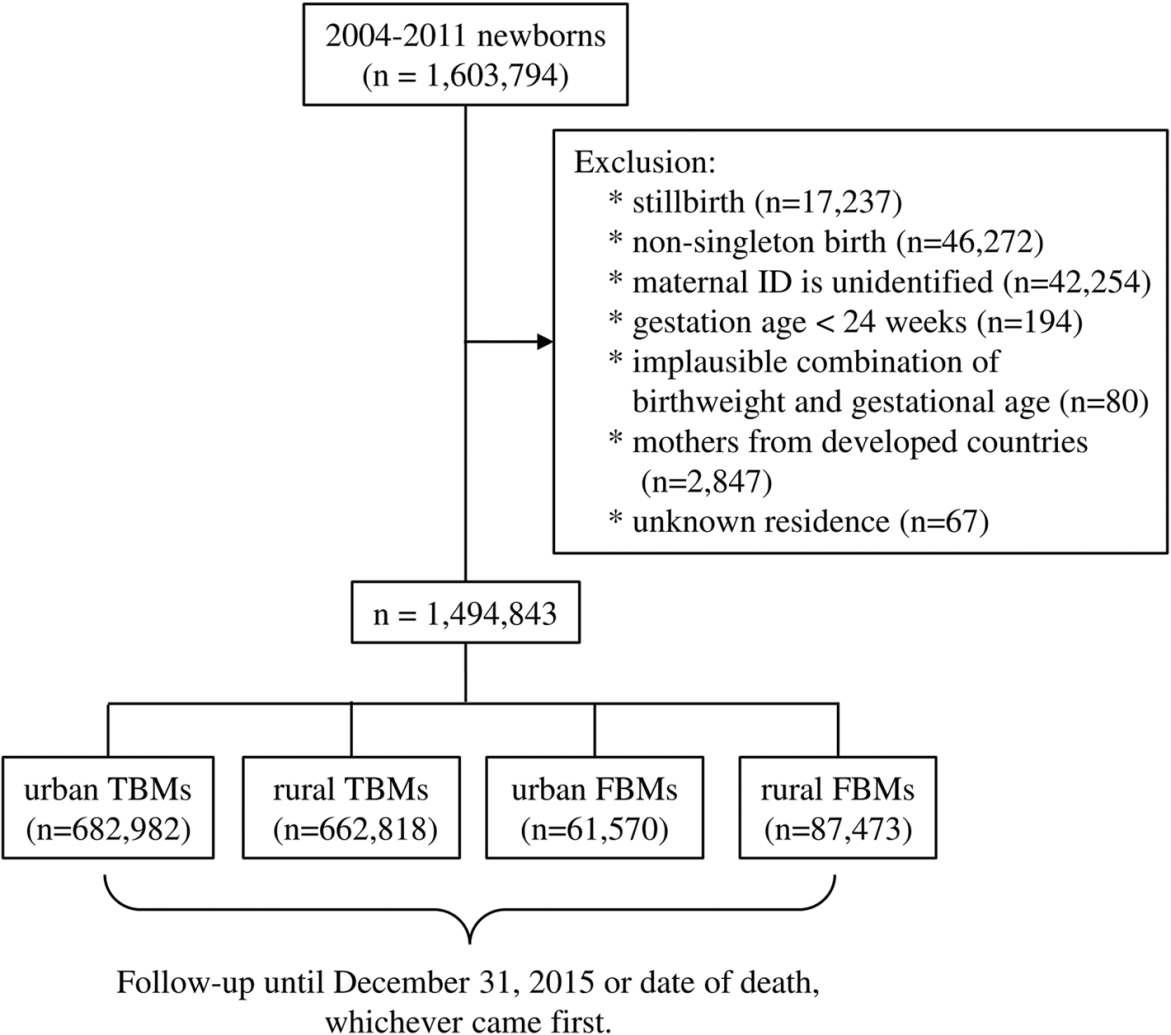
Flow chart of enrollment of the study subjects

A nationwide dynamic cohort, in which subjects are recruited at different times, was used. The advantages of such a cohort are that the number of subjects does not decline, or suffer from the problem of aging, over time [15]. A live birth during 2004–2011 was identified in the TBR and followed-up in the NHIRD or TDR until December 31, 2015 or the date of death, whichever occurred first (Fig. 1). Children of FBMs from developed countries such as Japan, Korea, South Africa, UK, and US, among others, were excluded, because they represented less than 0.5% of all births [7].

### Outcomes

Mortality and severe diseases were studied. To avoid coding errors and misclassification bias in NHIRD, patients with severe diseases eligible to a catastrophic disease certificate were enrolled. In Taiwan, patients with severe or rare diseases (30 categories) are eligible to a catastrophic disease certificate (CDC). Patients with CDC can waive for the copayments of outpatient and inpatient. Issuance of the certificate requires a physician diagnosis of a catastrophic disease and a formal review by the NHI Administration (NHIA) with additional documentation including genetic studies, laboratory data, diagnostic images, or pathology reports. (see Additional file 1 for the 30 catastrophic diseases). To avoid spare data bias, only severe diseases with more than 100 incident cases during the follow-up period were included in this study [16].

### Covariates

Four groups were studied: urban TBMs, rural TBMs, urban FBMs, and rural FBMs. TBMs or FBMs was based on the maternal nationality from TBR. TBMs were the mothers whose nationality were Taiwan; whereas FBMs were the mothers whose nationality were foreign countries. The classification of urban or rural was based on the township in household registration of the mothers when they gave birth of the study children. In Liu’s study [17], they included population density (people/km^2^), population ratio of people with college or above educational levels, population ratio of elder people over 65 years old, population ratio of people of agriculture workers and the number of physicians per 100,000 people and used the cluster analysis with squared Euclidean distance and Wald’s minimum variance method, to study the urbanization stratification of varied township in Taiwan. They identified 7 clusters for the 359 townships of Taiwan: highly urbanized town, moderate urbanization town, emerging town, general township, aging town, agricultural town, remote towns and villages. In this study, we categorized the first 2 clusters as “urban” and the remaining 5 clusters as “rural”, proposed by Liu et al. [17] We classified health status at birth as small for gestational age (a birth weight below the 10th percentile), appropriate for gestational age (a birth weight between the 10th and 90th percentile), and large for gestational age (a birth weight above the 90th percentile).

### Statistical analysis

Disease incidence rates were calculated by dividing the total number of children with severe diseases during follow-up by person-years at risk. The 95% CI of the disease incidence rate was computed based on the Poisson distribution because the n is large and the rate is very small here and the binomial distribution is very similar to Poisson distribution [18]. The chi-squared test, Fisher’s exact test, and analysis of variance (ANOVA) were used for univariable comparisons of differences among the 4 study groups, where appropriate. The log-rank test (univariable) and Cox proportional hazards model (multivariable) was used to examine differences among the 4 study groups and the reference group was rural TBMs. Other than the status of the study group, we also included the grouping of birthweight-gestational-age, sex, and maternal age in the Cox proportional hazard model, because we studied numerous health outcomes. When comparing urban and rural mothers, the reference group was rural mothers (rural TBMs and rural FBMs). When comparing TBMs and FBMs, the reference group was FBMs (urban and rural FBMs). SAS 9.4 was used for the statistical analysis. The significance level of the study was .05.

## Results

### Children and maternal characteristics

From 2004 through 2011, 1,603,794 infants were born. A total of 1,494,843 singleton live births were included after excluding stillbirths, non-singleton births, children with missing maternal ID numbers, premature newborns with a gestational age less than 24 weeks, implausible combinations of birth weight and gestational age, children of mothers from developed countries, and children with missing data on residence area. There were 682,982 (45.7%) singleton live births to urban TBMs, 662,818 (44.3%) to rural TBMs, 61,570 (4.1%) to urban FBMs, and 87,473 (5.9%) to rural FBMs. Regarding to nationality of FBMs, mainland China (52.94%) was the predominant country, followed by Vietnam (33.01%), Indonesia (7.29%), Philippines (2.24%), Thailand (1.94%), Cambodia (1.31%), Myanmar (1.28%) (Fig. 1).

The annual numbers of births from 2004 through 2011 declined in all 4 maternal groups, except for the sudden drop in 2010 which is Tiger year, a Chinese zodiac. In Chinese belief, people born in Tiger year tend to have an unlucky life [19]. The male:female ratio of 52:48 remained constant among the 4 maternal groups. The mean birth weight was lowest for rural TBMs, followed by urban TBMs, rural FBMs, and urban FBMs. The rate of low birth weight (< 2500 g), preterm rate (< 37 weeks) and percentage of SGA children was highest for rural TBMs, followed by urban TBMs, rural FBMs, and urban FBMs (Table 1).

**Table 1:** Demographic and birth characteristics of newborns in Taiwan, by maternal status

	Urban TBMs (*n* = 682,982)		Rural TBMs (*n* = 662,818)		Urban FBMs (*n* = 61,570)		Rural FBMs (*n* = 87,473)		*P*
	No.	(%)	No.	(%)	No.	(%)	No.	(%)	
(a) Children									<.0001
Birth year									
2004	89,070	(13.04)	92,131	(13.90)	10,036	(16.30)	16,024	(18.32)	
2005	86,030	(12.60)	87,534	(13.21)	9466	(15.37)	14,995	(17.14)	
2006	86,886	(12.72)	86,747	(13.09)	8838	(14.35)	13,069	(14.94)	
2007	89,043	(13.04)	86,924	(13.11)	8017	(13.02)	11,161	(12.76)	
2008	86,835	(12.71)	84,528	(12.75)	7396	(12.01)	9915	(11.33)	
2009	86,556	(12.67)	82,350	(12.42)	6539	(10.62)	8636	(9.87)	
2010	70,995	(10.39)	65,116	(9.82)	5355	(8.70)	6734	(7.70)	
2011	87,567	(12.82)	77,488	(11.69)	5923	(9.62)	6939	(7.93)	
Sex									0.0606
Male	356,481	(52.19)	346,345	(52.25)	32,260	(52.40)	46,068	(52.67)	
Female	326,501	(47.81)	316,473	(47.75)	29,310	(47.60)	41,405	(47.33)	
Birth weight (g)									
mean (SD)	(3113.6	±430.2)^a^	(3090.9	±433.1)^b^	(3185.6	±415.1)^c^	(3154.0	±410.4)^d^	<.0001
< 2500	39,116	(5.73)	41,935	(6.33)	2275	(3.69)	3545	(4.05)	<.0001
normal	629,974	(92.24)	608,332	(91.78)	57,558	(93.48)	81,939	(93.67)	
≥ 4000	13,892	(2.03)	12,551	(1.89)	1737	(2.82)	1989	(2.27)	
Gestational age (wk)									
(mean ± SD)	(38.41	±1.54)^a^	(38.35	±1.57)^b^	(38.66	±1.44)^c^	(38.60	±1.45)^d^	<.0001
Preterm (< 37 wk)	47,233	(6.92)^a^	50,349	(7.60)^b^	3077	(5.00)^c^	4829	(5.52)^d^	<.0001
Birth weight for gestational age ^e^									<.0001
SGA	65,520	(9.59)	69,723	(10.52)	4743	(7.70)	7508	(8.58)	
AGA	550,004	(80.53)	531,092	(80.13)	49,439	(80.30)	70,863	(81.01)	
LGA	67,458	(9.88)	62,003	(9.35)	7388	(12.00)	9102	(10.41)	
(b) Mothers									
Maternal age (yrs)									
(mean ± sd)	(30.79	±4.57)^a^	(28.96	±4.57)^b^	(27.48	±4.69)^c^	(26.69	±4.61)^d^	<.0001
< 20	7128	(1.04)	15,725	(2.37)	752	(1.22)	1631	(1.86)	<.0001
20–24	51,123	(7.49)	99,752	(15.05)	17,440	(28.33)	30,411	(34.77)	
25–29	199,751	(29.25)	248,871	(37.55)	24,303	(39.47)	33,486	(38.28)	
30–34	286,900	(42.01)	218,267	(32.93)	13,912	(22.60)	16,216	(18.54)	
≥ 35	138,080	(20.22)	80,203	(12.10)	5163	(8.39)	5729	(6.55)	
Employment									<.0001
No	157,012	(22.99)	169,162	(25.52)	50,167	(81.48)	72,149	(82.48)	
Yes	498,778	(73.03)	484,835	(73.15)	8602	(13.97)	11,803	(13.49)	
Missing	27,192	(3.98)	8821	(1.33)	2801	(4.55)	3521	(4.03)	
Monthly income ^f^									<.0001
< p20	159,098	(23.29)	171,260	(25.84)	50,212	(81.55)	72,205	(82.55)	
p20-p60	130,885	(19.16)	145,481	(21.95)	4754	(7.72)	7305	(8.35)	
> p80	365,807	(53.56)	337,256	(50.89)	3803	(6.18)	4442	(5.08)	
Missing	27,192	(3.98)	8821	(1.33)	2801	(4.55)	3521	(4.03)	

^a, b, c, d^ different letter represent significant difference among four materanl groups ^e^ *SGA* small for gestational age, *AGA* appropriate for gestational age, *LGA* large for gestational age

^f^ p20 (percentile 20) = TWD$15,840; p40 = TWD $19,200; p60 = TWD $21,900; p80 = TWD $34,800

Maternal age at delivery was mean age of 26.69 years for rural FBMs, 27.48 years for urban FBMs, 28.96 years for rural TBMs, and 30.79 years for urban TBMs. About 24.2% of TBMs were unemployed, whereas 82.1% of FBMs were unemployed. Similarly, most FBMs had an income less than p20 (NT$15840 per month) (Table 1).

### Incidence rates of 18 severe diseases

**Fig. 2:**
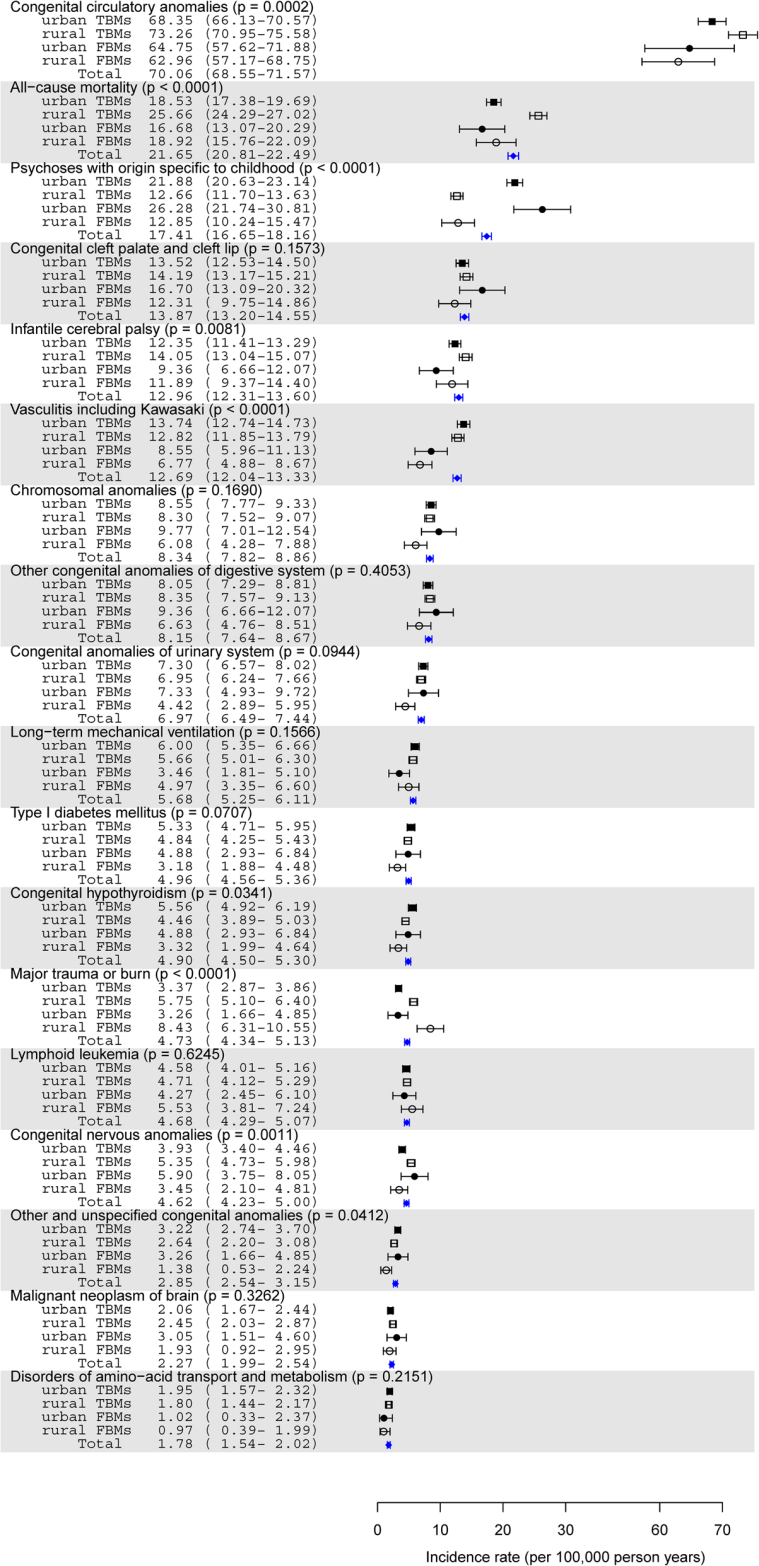
Incidence rates of 18 severe diseases of children among four maternal groups

Figure 2 ranks the incidence rates of 18 severe diseases for the 4 maternal groups. Congenital circulatory anomalies had the highest incidence rate, followed by all-cause mortality, psychoses with origin specific to childhood, congenital cleft palate and cleft lip, infantile cerebral palsy, vasculitis (in children, almost all Kawasaki disease), chromosomal anomalies, other congenital anomalies of the digestive system, congenital anomalies of the urinary system, long-term mechanical ventilation, type I diabetes mellitus, congenital hypothyroidism, major trauma/burn, lymphoid leukemia, congenital nervous anomalies, other and unspecified congenital anomalies, malignant neoplasm of the brain, and disorders of amino-acid transport and metabolism. The incidence rates of 8 severe diseases significantly differed among the 4 maternal groups, namely, congenital circulatory anomalies (*p* = .0002), all-cause mortality (*p* < .0001), psychoses (*p* < .0001), infantile cerebral palsy (*p* = .0081), vasculitis, including Kawasaki disease (*p* < .0001), congenital hypothyroidism (*p* = .0341), major trauma/burn (*p* < .0001), and congenital nervous anomalies (*p* = .0011). The category other and unspecified congenital anomalies was statistically significant (*p* = .0412) but was not considered because such events were combined from several congenital anomalies.

### Adjusted Hazard ratios for 8 severe diseases

**Fig. 3:**
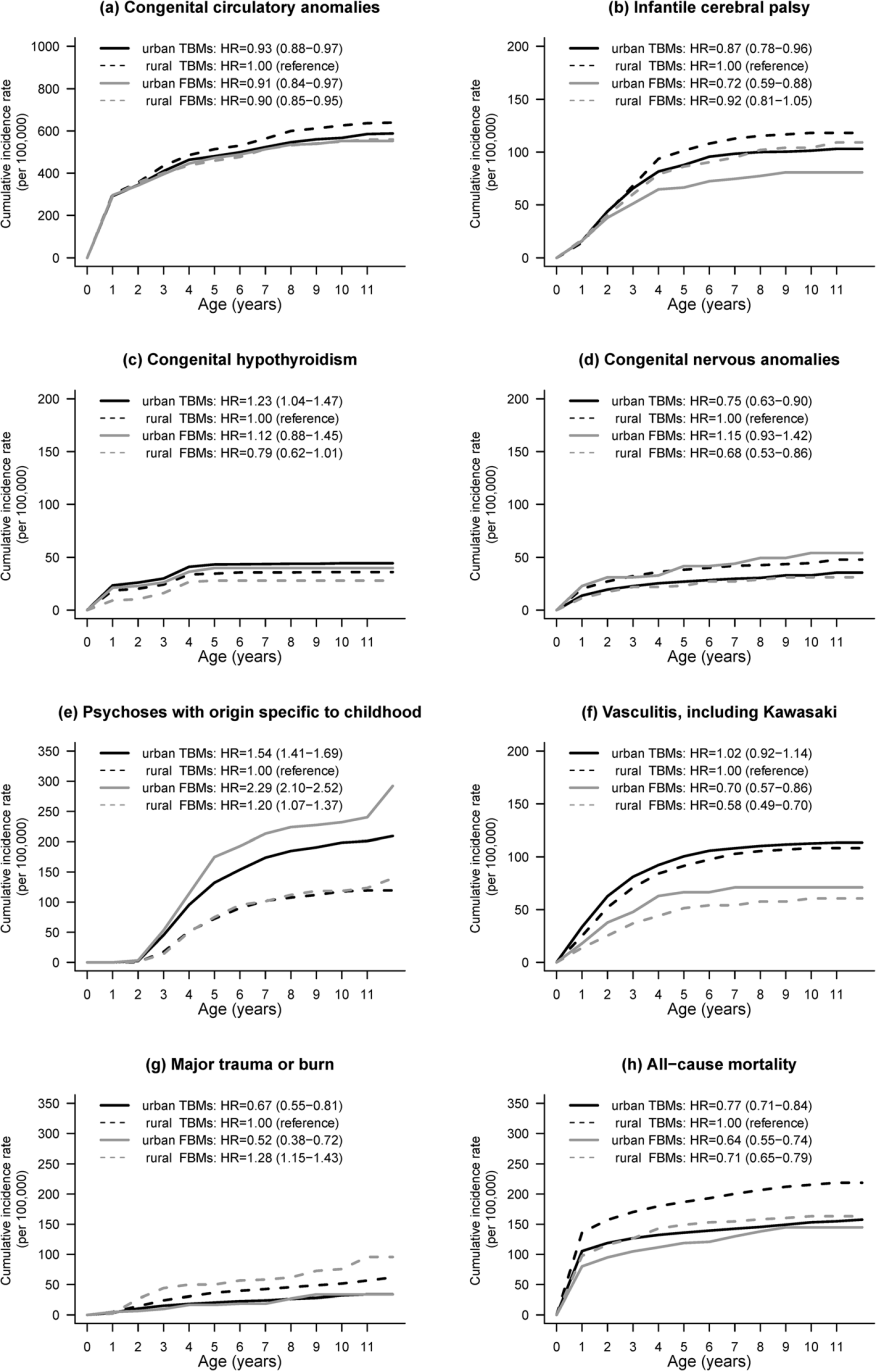
Cumulative incidence rates of eight severe diseases of children among four maternal groups. The hazard ratio was adjusted for the grouping of birthweight-gestational-age, sex, and maternal age using the Cox proportional hazard model

Figure 3 shows age-specific cumulative incidence rates and adjusted hazard ratios (HRs) for 8 severe diseases among children in the 4 maternal groups. Four major patterns were observed, as detailed below.

1. The incidence of vasculitis differed between FBMs and TBMs: the cumulative incidence rate was higher for children of TBMs than for children of FBMs (Fig. 3f). The adjusted HR for FBMs, as compared with TBMs, was 0.63 (95% CI, 0.50–0.78).
2. Incidence rates for several conditions differed between urban and rural mothers, namely, congenital hypothyroidism (Fig. 3c), psychoses (Fig. 3e), major trauma/burn (Fig. 3g), and all-cause mortality (Fig. 3h). Rates of congenital hypothyroidism and psychoses were higher among urban children than among rural children. As compared with urban children, rural children had adjusted HRs of 0.80 (95% CI, 0.67–0.94) for congenital hypothyroidism and 0.64 (95% CI, 0.58–0.70) for psychoses. In contrast, rates of major trauma/burn and all-cause mortality were higher in rural areas than in urban areas. In rural areas, the adjusted HRs were 1.59 (95% CI, 1.33–1.90) for major trauma/burn and 1.28 (95% CI, 1.18–1.38) for all-cause mortality, as compared with urban areas.
3. Rates of congenital circulatory anomalies (Fig. 3a) and infant cerebral palsy (Fig. 3b) were highest among the children of rural TBMs. As compared with rural TBMs, the adjusted HR for congenital circulatory anomalies was 0.93 (95% CI, 0.89–0.97) for urban TBMs, 0.91 (95% CI, 0.81–1.02) for urban FBMs, and 0.90 (95% CI, 0.81–0.99) for rural FBMs. As compared with rural TBMs, the adjusted HR for infant cerebral palsy was 0.87 (95% CI, 0.78–0.97) for urban TBMs, 0.72 (95% CI, 0.53–0.97) for urban FBMs, and 0.92 (95% CI, 0.74–1.15) for rural FBMs.
4. Incidence rates did not greatly differ between the children of FBMs and TBMs, but there was a variable urban—rural difference in congenital anomalies of the nervous system (Fig. 3d). Although children of urban FBMs had a higher cumulative incidence rate of congenital nervous anomalies than did children of urban TBMs, the relationship reversed among children of rural mothers.

### Age

Incidence rates varied with age (Fig. 3). The cumulative incidence rates of congenital circulatory anomalies, congenital hypothyroidism, and congenital nervous anomalies increased quickly until age 1 year and increased more slowly until age 5 years. The cumulative incidences of infantile cerebral palsy and vasculitis rapidly increased until age 5 years and remained relatively constant thereafter. No child developed psychosis before age 2 years, but rates rose sharply, and differed in relation to urbanicity, after that. Incidence rates for major trauma/burn and all-cause mortality exhibited a similar pattern, except that rates rose slowly after age 18 months.

## Discussion

In this study, we saw a better outcome of birth weight and gestational age of children of FBMs than those of TBMs, consistent with previous studies [1, 7, 11, 20]. The better birth outcomes for newborns of FBMs can be explained in part by the healthy migrant effect [2, 11]. However, we noted no such effect on congenital anomalies of 18 severe diseases. In this study, children of FBMs had a lower incidence of vasculitis, mainly Kawasaki disease. Kawasaki disease is most prevalent between the ages of 6 months and 5 years in Northeast Asia, and is less seen in Southeast Asia [21]. Genetic of host susceptibility may be an explanation of the lower incidence rate of Kawasaki among children of FBMs. Recent genome-wide studies of Kawasaki disease have identified several gene variants and mutations, but the impact of genetic factors on disease susceptibility in different ethnic groups awaits further investigation.

We identified several urban–rural disparities in child health. Most importantly, urban children had a higher incidence of psychotic disorders, agree with a previous study [22]. High population density, noise, stressful life events in the family, and diminished social support may influence psychosis development in both adults and children [23–25]. We noted 2 peaks in the cumulative incidence curve for mental illness: one during the preschool period and a potential second peak during adolescence. For children and adolescents, acculturation can be stressful, and this stress is associated with mental health status [26]. Language and cultural differences between parents may affect mental development during early childhood [27], and cultural identity might have effects on integration and friendships during school age [28].

In this study, children in rural areas had a higher incidence of major trauma/burn and all-cause mortality, most likely due to insufficient knowledge and inadequate attention to child safety and injury prevention. The low educational level of the rural population [29], the low density of pediatricians, and the limited availability of medical resources [30] have adverse effects on infant and child mortality. Comprehensive NHI coverage of children in Taiwan has not addressed urban–rural differences in health status, and more efforts are needed to improve access to medical resources, as well as attitudes and actions regarding child safety and injury prevention.

Children born to rural TBMs had the highest incidence rates of congenital circulatory anomalies and infantile cerebral palsy. Congenital heart defects are the most common birth defects in Taiwan [31]. Previous studies revealed ethnic disparities in incidence [32–34]. However, we found no significant differences between TBMs and FBMs. Instead, children of rural TBMs had the highest incidence, perhaps because of the higher prevalence of alcohol misuse in rural areas [35]. In addition, the law in Taiwan allows abortion for reasons of fetal, maternal, or social factors, if it is performed prior to 24 gestational weeks. Better accessibility of level II prenatal sonography in urban areas and selective termination of pregnancies in which the fetus exhibits major congenital circulatory anomalies before gestational age 24 weeks might partly explain the lower incidences of these conditions in urban areas. Unfortunately, the annual termination rate in Taiwan is unavailable as the notification of artificial abortion before 24 weeks of gestation to the health authority is not mandatory. We were unable to show the difference in termination rate between urban and rural areas.

The introduction of vaccines, antibiotic medications, and antimicrobial disinfectants explains why no infectious disease was included among the 18 severe diseases of children in Taiwan. Most severe diseases were related to congenital disorders. Thus, the cumulative incidences of congenital diseases increased rapidly in infants and began to decrease in early childhood, consistent with the onset of clinical manifestations for the diseases.

The zero cumulative incidence of psychoses for children younger than 2 years is probably due to the difficulty of evaluating the mental status of this age group. Our findings revealed a drastic increase in the cumulative mortality rate before age 1 year, and the average under-5 mortality rate was 1.3 per 1000. The present mortality rate is similar to the updated under-5 mortality rate reported by the Global Burden of Disease Study 2013 [36]. Deaths before age 1 year still account for most all-cause mortality, as in previous reports [37, 38].

### Strengths and limitations

The longitudinal design allowed us to evaluate child health from birth until age 11 years. Severe diseases based on catastrophic diseases, which was carefully validated by physicians and reviewed by committee, minimized the problem of misclassification. However, children with mild physical diseases or mental disorders are not included. Hence, the incidence rates of the 18 severe diseases in this study tended to be lower than those reported previously. For instance, the incidence of congenital circulatory anomalies was 70.06 per 100,000 person-years in this study, but the incidence of congenital heart diseases in Taiwan was 13.08 per 1000 live births [39]. We anticipated that bigger effect of FBM/TBM or urban/rural on children’s mild health outcome. We did not examine how parental health status affects child health because maternal disease before or during pregnancy was not always recorded in the TBR. Future studies should review medical records to examine this issue. Furthermore, frequency of parental tobacco use and alcohol use disorders was not studied because lifestyle data were not available. Finally, the status of urban and rural was based on household registration of the mothers when they gave birth of the study children. We did not consider children who moved from urban to rural area or vise versa. Hence, for diseases with very different incidence rates between urban and rural area, such as congenital hypothyroidism, psychoses, etc., we suggest that the status of urban and rural should be treated as time-varying rather than static in future studies.

## Conclusions

Except for vasculitis, we did not observe a healthy migrant effect in the incidence rates of 18 severe diseases in this study. Increased attention should be focused on the mental health of children in urban areas. Despite broad NHI coverage, the risks of major trauma/burn and all-cause mortality were greater for rural children than for urban children.

## Supplementary information

**Additional file 1** Catastrophic Diseases in the Taiwan National Health Insurance System.

## Data Availability

The datasets used during the current study are from Health and Welfare Data Science Center (HWDC), Ministry of Health and Welfare (MOHW), Taiwan. All datasets are only available for on-site analysis and from the authors upon reasonable request and with permission.

## Abbreviations

AGA: appropriate for gestational age
CDC: catastrophic disease certificate
CI: confidence interval
FBMs: foreign-born mothers
HRs: hazard ratios
LBW: low birth weight
LGA: large for gestational age
NHI: national health insurance
NHIRD: national health insurance research database
SGA: small for gestational age
TBMs: Taiwan-born mothers
TBR: Taiwan birth registry
TDR: Taiwan death registry
