# Supplementary material for: Association of maternal ethnicity and urbanicity on severe pediatric disease: a nationwide cohort study
Source: BMC Pediatr. 2019 Dec 23;19:514. doi: 10.1186/s12887-019-1885-9 (PMC6927117; doi:10.1186/s12887-019-1885-9)
Supplement: Supplementary file 1 — Additional file 1 Catastrophic Diseases in the Taiwan National Health Insurance System. [file 12887_2019_1885_MOESM1_ESM.docx]

**Catastrophic illness in National Health Insurance program, Taiwan**

| **Catastrophic illness** | ICD-9-CM codes | catastrophic illness certificate (CIC) valid period |
| --- | --- | --- |
| **1. Malignant neoplasm requiring active or long-term treatment** | 140-208 | 5 years |
| **2. Hereditary coagulation factor deficiency** |  | Permanent |
| 2.1 Hereditary factor VIII deficiency | 286.0 |  |
| 2.2 Hereditary factor IX deficiency | 286.1 |  |
| 2.3 Hereditary factor XI deficiency | 286.2 |  |
| 2.4 Hereditary deficiency of other clotting factors | 286.3 |  |
| **3. Severe hemolytic anemias and aplastic anemias (untreated hemoglobin, usually Hb <8 gm/dl for adult or <12 gm/dl for neonate)** |  | 5 years |
| 3.1 Hereditary hemolytic anemias | 282 |  |
| 3.2 Acquired hemolytic anemias | 283 |  |
| 3.3 Aplastic anemias | 284 |  |
| **4. Chronic Renal failure (Uremia), requiring regular dialysis therapy** |  | Permanent: Upon application the need for regular dialysis treatment is determined.  3 months: Upon application the need for regular dialysis treatment is to be determined. |
| 4.1 Chronic kidney disease | 585 |  |
| 4.2 Hypertensive chronic kidney disease with stage 5 chronic kidney disease or end stage renal disease | 403.01, 403.11, 403.91 |  |
| 4.3 Hypertensive heart and chronic kidney disease with stage 5 chronic kidney disease or end stage renal disease | 404.02, 404.03, 404.12, 404.13, 404.92, 404.93 |  |
| **5. Systemic autoimmune syndrome requiring life-long treatment** |  | Permanent |
| 5.1 Systemic lupus erythematosus | 710.0 |  |
| 5.2 Systemic sclerosis | 710.1 |  |
| 5.3 Rheumatoid arthritis (Rheumatoid arthritis juvenile) | 714.0, 714.30~714.33 |  |
| 5.4 Polymyositis | 710.4 |  |
| 5.5 Dermatomyositis | 710.3 |  |
| 5.6 Vasculitis |  |  |
| 5.6.1 Polyarteritis nodosa | 446.0 |  |
| 5.6.2 Hypersensitivity angiitis | 446.2 |  |
| 5.6.3 Wegener’s granulomatosis | 446.4 |  |
| 5.6.4 Giant cell arteritis | 446.5 |  |
| 5.6.5 Thromboangiitis obliterans (Buerger’s disease) | 443.1 |  |
| 5.6.6 Aortic arch syndrome (Takayasu) | 446.7 |  |
| 5.6.7 Kawasaki disease | 446.1 |  |
| 5.6.8 Behcet’s disease | 136.1 |  |
| 5.7 Pemphigus | 694.4 |  |
| 5.8 Sjogren’s syndrome | 710.2 |  |
| 5.9 Crohn’s disease | 555 |  |
| 5.10 Ulcerative colitis | 556.0~556.6, 56.8~556.9 |  |
| **6. Chronic mental disorders (referring to diagnoses listed below with confirmed chronicity. Except for 6.1 dementia, diagnosis of any following disorder requires a certificate issued by a board certified psychiatrist with his specialist license number identified in the certificate)** |  |  |
| 6.1 Unspecified dementia (organic psychotic conditions) (diagnosis of this chronic mental disorder requires a medical certificate issued by a board certified psychiatrist or neurologist with his specialist license number identified in the certificate) | 290 | Permanent |
| 6.2 Subacute delirium | 293.1 | 6 months (Re-evaluation required every six months) |
| 6.3 Other organic psychotic conditions (chronic) | 294 | 2 years: initial issue  Permanent: renewal |
| 6.4 Schizophrenic disorders | 295 | Permanent |
| 6.5 Affective psychoses | 296 | 2 years: initial issue |
| 6.6 Paranoid states | 297 | Permanent: renewal |
| 6.7 Psychoses with origin specific to childhood | 299 |  |
| 6.7.1 Infantile autism | 299.0 | 5 years: initial issue  Permanent: renewal |
| 6.7.2 Disintegrative psychoses | 299.1 | 5 years: initial issue  Permanent: renewal |
| 6.7.3 Other specified early childhood psychoses | 299.8 | 5 years: initial issue  Permanent: renewal |
| 6.7.4 Psychoses with origin specific to childhood unspecified | 299.9 | 3 years: first renewal;  5 years: second renewal;  Permanent: after the third renewal |
| **7. Congenital metabolic disorders (excluding G6PD metabolic disorders)** |  | Permanent |
| 7.1 Congenital hypothyroidism | 243 |  |
| 7.2 Type I diabetes mellitus | 250.01, 250.03, 250.11, 250.13, 250.21, 250.23, 250.31, 250.33, 250.41, 250.43, 250.51, 250.53, 250.61, 250.63, 250.71, 250.73, 250.81, 250.83, 250.91, 250.93, |  |
| 7.3 Diabetes insipidus | 253.5 |  |
| 7.4 Congenital adrenal hyperplasia | 255.2 |  |
| 7.5 Disorders of amino-acid transport and metabolism | 270 |  |
| 7.6 Glycogent storage disease | 271.0 |  |
| 7.7 Galactosemia | 271.1 |  |
| 7.8 Pure hyperglyceridemia | 272.1 |  |
| 7.9 Lipodystrophy | 272.6 |  |
| 7.10 Lipidoses | 272.7 |  |
| 7.11 Disorders of lipoid metabolism | 272.9 |  |
| 7.12 Disorders of copper metabolism | 275.1 |  |
| 7.13 Disorders of calcium metabolism | 275.40~275.42, 275.49 |  |
| 7.14 Other disorders of purine and pyrimidine metabolism | 277.2 |  |
| 7.15 Mucopolysaccharidosis | 277.5 |  |
| 7.16 Other specified disorders of metabolism | 277.8 |  |
| 7.17 Disorders of metabolism | 277.9 |  |
| **8. Congenital and chromosomal abnormalities of heart, lung, digestive system, kidney, neurological and skeletal systems** |  |  |
| 8.1 Anencephalus and similar anomalies | 740 | Permanent |
| 8.2 Other congenital anomalies of nervous system | 742 | 3 years |
| 8.3 Bulbus cordis anomalies and anomalies of cardiac septal closure or other congenital anomalies of heart | 745~746 | 3 years |
| 8.4 Other congenital anomalies of circulatory system | 747 | 3 years |
| 8.5 Congenital cystic lung disease | 748.4 | Permanent |
| 8.6 Agenesis, hypoplasia and dysplasia of lung | 748.5 | Permanent |
| 8.7 Other anomalies of lung | 748.6 | Permanent |
| 8.8 Other congenital anomalies of digestive system | 751 | Permanent |
| 8.9 Renal agenesis and dysgenesis | 753.0 | Permanent |
| 8.10 Cystic kidney disease | 753.1 | Permanent |
| 8.11 Obstructive defects of renal pelvis and ureter | 753.20~753.23, 753.29 | Permanent |
| 8.12 Other specified anomalies of kidney | 753.3 | Permanent |
| 8.13 Chondrodystrophy | 756.4 | Permanent |
| 8.14 Chromosomal anomalies | 758 | Permanent |
| 8.15 Congenital cleft palate and cleft lip | 749.01~749.04, 749.11~749.14, 749.21~749.25 | 3 years |
| **9. Burn of >20% of total body surface or facial burn with facial organ functional impairment** |  | 1 year |
| 9.1 Burn of >20% of total body surface | 948.2~948.9 |  |
| 9.2 Facial burn with facial organ functional impairment |  |  |
| 9.2.1 Burn confined to eye and adnexa | 940 |  |
| 9.2.2 Burn of face and head, deep necrosis of underlying tissue (deep third degree) with loss of a body part | 941.5 |  |
| **10. Follow up managements after kidney, heart, lung, liver, bone marrow, or pancreas transplantation** |  |  |
| 10.1 Kidney replaced by transplant | V42.0 | Permanent |
| 10.2 Heart replaced by transplant | V42.1 | Permanent |
| 10.3 Lung replaced by transplant | V42.6 | Permanent |
| 10.4 Liver replaced by transplant | V42.7 | Permanent |
| 10.5 Bone marrow replaced by transplant | V42.81~V42.82 | 5 years |
| 10.6 Pancreas replaced by transplant | V42.83 | Permanent |
| 10.7 Intestines replaced by transplant | V42.84 | Permanent |
| 10.8 Complication of transplanted kidney | 996.81 | Permanent |
| 10.9 Complication of transplanted liver | 996.82 | Permanent |
| 10.10 Complication of transplanted heart | 996.83 | Permanent |
| 10.11 Complication of transplanted lung | 996.84 | Permanent |
| 10.12 Complication of transplanted bone marrow | 996.85 | 5 years |
| 10.13 Complication of transplanted pancreas | 996.86 | Permanent |
| 10.14 Complications of transplanted intestine | 996.87 | Permanent |
| **11. Late effects of acute poliomyelitis or cerebral palsy in neurological, musculoskeletal or pulmonary system with at least moderate degree of disability** |  | Permanent |
| 11.1 Acute poliomyelitis with other paralysis | 045.1 |  |
| 11.2 Infantile cerebral palsy | 343 |  |
| 11.3 Other paralytic syndromes late effects of acute poliomyelitis | 344+138 |  |
| **12. Major trauma rated 16 or above on the severity scale (injury severity score ≥16) (ISS is not applicable to patients under vegetative status)** | 959.99 | 1 year: initial issue;  3 years: renewal |
| **13. Long-term mechanical ventilation, defined as one of the following:** | 518.85 | 42 days: initial issue;  3 months: renewal;  1 year: after the second renewal |
| 13.1 Invasive mechanical ventilation for 21 or more days. |  |  |
| 13.2 Invasive mechanical ventilation followed by non-invasive ventilation, with a total duration of 21 or more days. |  |  |
| 13.3 Invasive mechanical ventilation followed by negative pressure ventilation, with a total duration of 21 or more days. |  |  |
| 13.4 Specific diseases, e.g., End stage heart failure, chronic pulmonary diseases, primary neuromuscular diseases, chronic hypoventilation syndrome, which require non-invasive ventilation for 21 or more days. |  |  |
| **14.** |  | 3 months: initial issue;  3 years: renewal |
| 14.1 Patients suffering from severe malnutrition due to major enterectomy, intestinal failure already on a fully intravenous diet for 30 days, and unable to obtain sufficient nutrition through an oral diet | 261.0 |  |
| 14.2 Patients suffering from severe malnutrition due to other chronic disease already on a fully intravenous diet for 30 days, and unable to obtain sufficient nutrition through an oral diet | 261.1 |  |
| **15. Severe decompression sickness or air embolism caused by diving or improper decompression, along with respiratory, circulatory or nervous system complications and requires long-term treatment** |  |  |
| 15.1 Decompression sickness | 993.3 | Permanent |
| 15.2 Air embolism | 958.0 | 3 years |
| **16. Myasthenia gravis** | 358.0 | 3 years |
| **17. Congenital immunodeficiency disorders** |  | 5 years |
| 17.1 Hypogammaglobulinemia | 279.00, 279.06 |  |
| 17.2 Selective immunoglobulin deficiency combined with repeated related infection | 279.08 |  |
| 17.3 Deficiency of cell-mediated immunity | 279.1 |  |
| 17.4 Combined immunity deficiency | 279.2 |  |
| 17.5 Phagocyte deficiency (chronic granulomatous disease) | 279.3 |  |
| 17.6 Other specified disorder of immune mechanism | 279.8 |  |
| **18.Spinal cord injury or pathology causing neurological, musculoskeletal, dermatological, cardiopulmonary, urogenital, and gastroenteral system complications with at least moderate degree of disability** |  | Permanent |
| 18.1 Fracture of vertebral column with spinal cord injury | 806 |  |
| 18.2 Spinal cord injury without evidence of spinal bone injury | 952 |  |
| 18.3 Other disease of spinal cord | 336 |  |
| **19. Occupational disease (Referring only to the occupational diseases listed on the Occupational Diseases List (Exhibit 1) under Article 34, Paragraph 1, of the Labor Insurance Act. Eligibility covers only National Health Insurance (NHI) beneficiaries who are retired and not insured persons under the Labor Insurance Program (LIP). NHI beneficiaries with any of these occupational diseases while being insured persons under the LIP should follow the regulations set forth under the LIP when seeking medical attention, and part of their NHI co-payment is exempted.)** |  | 3 years: initial issue;  Permanent: renewal |
| 19.1 Coal workers’ pneumoconiosis | 500 |  |
| 19.2 Asbestosis | 501 |  |
| 19.3 Pneumoconiosis due to other silica or silicates | 502 |  |
| 19.4 Pneumoconiosis due to other inorganic dust | 503 |  |
| 19.5 Pneumoconiosis | 505 |  |
| **20. Cerebrovascular disease (acute stage, limited to within one month of the acute attack)** |  | Directly identified by the physician within one month after the acute attack.  No need to apply for a certificate. |
| 20.1 Subarachnoid hemorrhage | 4304 |  |
| 20.2 Intracerebral hemorrhage | 431, 432 |  |
| 20.3 Cerebral infarction | 433, 434 |  |
| 20.4 Other cerebrovascular disease | 435~437 |  |
| **21. Multiple sclerosis** | 340 | 5 years |
| **22. Congenital muscular dystrophy** | 359.0、359.1 | Permanent |
| **23. Congenital anomalies integument** |  | Permanent |
| 23.1 Congenital epidermolysis bullosa | 757.39 |  |
| 23.2 Congenital malformation of integument, unspecified | 757.9 |  |
| 23.3 Congenital Ichthyosis | 757.1 |  |
| **24. Leprosy (Hansen’s disease)** | 030 | Permanent |
| **25. Liver cirrhosis with complication** | 571.2, 571.5, 571.6 | 5 years |
| 25.1 Ascites with poor control |  |  |
| 25.2 Esophageal or gastric varicosis bleeding |  |  |
| 25.3 Hepatic coma or liver dyscompensated |  |  |
| **26. Neurological, muscular, skeletal, cardiac or pulmonary complications of premature infants** |  |  |
| 26.1 Neurological, muscular, skeletal, cardiac or pulmonary complications due to premature infants to have admission care within three months birth. | 765.90 | Directly identified by the physician. No need to apply for a certificate. |
| 26.2 Neurological, muscular, skeletal, cardiac or pulmonary complications due to premature infants certified to have moderate impairments three months of age. | 765.99 | 3 years |
| **27. Toxic effect of arsenic and its compounds (black foot disease)** | 985.1 | Permanent |
| **28. Motor neuron disease with at least moderate degree of disability or requiring long-term ventilator use (This restriction dose not apply to patients diagnosed with AMYOTROPHIC LATERAL SCLEROSIS (ICD-9-CM 335.20) by neurologists)** | 335.2 | Permanent |
| **29. Creutzfeldt-Jakob disease** | 046.1 | Permanent |
| **30. Rare disease (Other rare diseases not included in the 29 categories above, but were announced by the Ministry of Health and Welfare)** |  | Permanent |
